# Vitamin D Supplementation in Functional Recovery of Subjects with Severe Acquired Brain Injury: A Pilot Controlled Randomized Study

**DOI:** 10.1089/neur.2023.0128

**Published:** 2024-07-01

**Authors:** Domenico Intiso, Antonello Marco Centra, Michele Gravina, Massimiliano Copetti, Andrea Fontana, Michelangelo Bartolo, Serena Filoni, Filomena Di Rienzo

**Affiliations:** ^1^ Unit of Neuro-rehabilitation, and Rehabilitation Medicine, IRCCS “Casa Sollievo della Sofferenza,” San Giovanni Rotondo, Foggia, Italy.; ^2^ Unit of Biostatistics, IRCCS “Casa Sollievo della Sofferenza,” San Giovanni Rotondo, Foggia, Italy.; ^3^ Department of Rehabilitation, Neurorehabilitation Unit, Habilita Care & Research, Zingonia, Italy.

**Keywords:** acquired brain injury, outcome, rehabilitation, vitamin D, vitamin D supplementation

## Abstract

Low vitamin D (VD) has been associated with poor clinical course in several neurological diseases. Supplementation has been suggested to improve outcomes. Severe acquired brain injury (sABI) subjects have low VD levels and disabling conditions requiring rehabilitation. The aim of the present study was to evaluate if VD supplementation produced a better clinical course and a better functional outcome in sABI during rehabilitation. A randomized single-blind study was performed. sABI subjects were randomized to the VD supplementation group (VDsG) (initial dose of 50.000 UI and 1.000 daily) and usual care control group (CG). Disability Rating Scale (DRS), Glasgow Outcome Scale (GOS), and Level of Cognitive Functioning (LCF) were used in assessing disability. A total of 73 subjects (42 M and 31 F; mean age 53.2 ± 15.7) were randomized: 36 (21 M and 15 F; mean age 57.52 ± 14.88) to VDsG and 37 (20 M and 17 F; mean age 48.28 ± 17.47) to CG. Both groups significantly improved after rehabilitation, and no between-group difference was observed. The mean score values for DRS, GOS, and LCF in VDsG were 18.83 ± 4.27 and 9.42 ± 5.83; 2.89 ± 0.32 and 3.78 ± 0.80; and 4.81 ± 1.70 and 7.53 ± 1.28, at admission and discharge, respectively. Likewise, mean values for DRS, GOS, and LCF in CG were 18.57 ± 4.80 and 9.84 ± 6.34; 2.84 ± 0.37 and 3.81 ± 0.94; and 4.97 ± 2.01 and 7.41 ± 1.32, respectively. VD supplementation did not improve functional outcomes in sABI during rehabilitation treatment.

## Introduction

A number of studies have demonstrated the key role of 25-hydroxyvitamin D (VD) in physiological processes and the development of different organs and systems other than skeletal apparatus and phosphorus–calcium homeostasis. The action has been linked to several biological processes, such as differentiation and proliferation, immune modulation, and cell growth. In the last decade, it has been reported that deficiency of VD can be associated with several neurological diseases, including multiple sclerosis (MS),^1–3^ Parkinson’s disease,^4,5^ Alzheimer’s disease,^6,7^ spinal cord injury,^8^ and stroke.^9^ Furthermore, studies have reported that low serum levels of VD can promote poor prognosis and outcome in subjects suffering from many neurological disorders,^10^ particularly in those with ischemic stroke.^11–13^ Given the pleiotropic effects and the role of VD, its supplementation has been proposed to promote and improve the course in subjects who have deficiency of this element.^14^ Likewise, supplementation has been suggested in subjects with deficiency of VD particularly in patients with MS and ischemic stroke. In this respect, studies have demonstrated that VD supplementation can produce various benefits such as reduction of relapses in subjects with relapsing-remitting MS (RRMS) and improvement of outcome in stroke.^15–17^ Low VD levels have been found in traumatic brain injury patients undergoing acute inpatient rehabilitation,^18^ and a correlation between disability and low serum levels of VD has been detected in subjects with severe acquired brain injury (sABI).^19^ This neurological condition embraces a number of different neurological disturbances, including traumatic brain injury, hypoxic brain injury, stroke, and brain tumor, which produce a disorder of consciousness graded from 3 to 8 according to the Glasgow Coma Scale. Subjects with sABI suffer from cognitive, communicative, physical, emotional, or behavioral impairments^20,21^ that require intensive rehabilitation.

Recently, it has been detected that VD supplementation could improve the functional outcome in poststroke subjects undergoing rehabilitation.^17,22^ As subjects with sABI can have deficiency of VD, we hypothesized that its supplementation might favor the clinical and functional outcome. The aim of the present study was to evaluate if VD supplementation in subjects with sABI who underwent intensive rehabilitation produced a better clinical course and a better functional outcome.

## Method and Participants

With the approval of the Local Ethics Committee (ICF: V1_06 Ago 2020), consecutive adult subjects >18 years old with sABI admitted to neurorehabilitation from the intensive care unit (ICU) during the period December 2020–June 2023 were prospectively enrolled and randomized. Patients were excluded if (1) diagnosis was not in accordance with sABI; (2) subjects had previous cerebral lesions or pre-existent neurological diseases; (3) subjects had renal or liver failure; (4) patients had malignancy; (5) subjects had pre-existent VD supplementation; and (6) subjects had ascertained phosphorus–calcium disorders. Patients with normal serum levels of VD were not excluded. Patients provided informed consent in cases in which they were able to. In the cases in which the patient was incapable, the patient’s nearest relative provided consent for inclusion.

## Study Design

Subjects were randomized with a 1:1 ratio using a random number generator that was blinded to the patients and investigators. The randomization sequence was generated using SPSS, 20.0 version before the trial. The researcher responsible for randomization was independent of the assessor. Subjects randomized to VD treatment received an initial dose of 50.000 UI of VD, and then a daily oral formulation of VD (cholecalciferol-D3) at a dosage of 1.000 UI. All subjects randomized to the treatment received the dose of VD regardless of the basal assessed level dosage. Tablet or drops were used according to the clinical conditions of subjects. Because about all patients had severe dysphagia requiring nasogastric tube (NGT) or percutaneous endoscopic gastrostomy (PEG), drop formulations were used at admission until the patients recovered deglutition and oral feeding. Control group (CG) received usual care without VD supplementation.

Functional evaluation was assessed by means of the Disability Rating Scale (DRS),^23^ the Glasgow Outcome Scale (GOS),^24^ and The Rancho Level of Cognitive Functioning (LCF).^25^ The GOS provides a quantification of global functional recovery and dependence. It ranges from 0 to 5, with 0 representing a totally dependent bedridden state and 5 indicating that the patient is fully independent. The DRS stratifies patients with respect to disability and independence into the following four categories: no disability to moderate disability (range 0–6); moderately severe to severe disability (range 7–13); severe to extremely severe disability (range 14–21); and vegetative state to extreme vegetative state (range 22–29). It includes a fifth category: 30, death. LCF scale is one of the earlier developed scales used to assess cognitive functioning in postcoma patients. It ranges from 1 to 8, with 1 indicating no response and 8 indicating that the subject is appropriate and purposeful. Clinical and functional evaluations were performed for all subjects at admission and discharge by the same examiner blinded to treatment.

### VD dosage and blood parameter assessment

Serum levels of 25(OH)D were analyzed using a 25-hydroxy chemiluminescent immunoassay (DiaSorin Liaison; Stillwater, MN), which has 100% cross-reactivity with both metabolites, within 2 days from admission and at discharge. The serum level of 25(OH)D was stratified as sufficient (≥30.0 ng/mL [75 nmol/L]), insufficient (20.0–29.9 ng/mL [50–75 nmol/L]), and deficient (<20.0 ng/mL [50 nmol/L]) according to the Endocrine Society criteria.^26^ Blood parameters, including electrolytes (calcium and phosphorus), proteins, albumin, gamma-glutamyl transferase, alkaline phosphatase, and creatinine serum levels, were assessed at admission. Blood samples were collected in tubes with potassium ethylenediaminetetraacetic acid and analyzed 1 h after venipuncture.

### Rehabilitation interventions

All patients underwent an individualized rehabilitation treatment of 1–2 h daily, 6 days a week, according to their clinical conditions. The rehabilitation treatment consisted of joint mobilization, proprioceptive neuromuscular facilitation according to neurodevelopmental techniques, and flexibility and strength exercises. Further interventions aimed to remove tracheostomy devices, PEG, or NGT, and respiratory exercise, speech therapy, occupational therapy, and psychological support to patients, family, and/or caregivers were given when needed according to a rehabilitative team approach.

### Sample size calculation

A sample size of 70 patients (35 patients per arm) achieves 80% power with a type I error (alpha) of 0.05 to detect a statistically significant Cohen effect size of 0.7 when comparing functional changes between the two arms.

## Statistical Analyses

Patient characteristics were reported as mean ± standard deviation or median, along with the first–third quartiles (q1–q3) as appropriate, and as frequencies and percentages for continuous and categorical variables, respectively. Comparisons between groups were assessed using the Mann–Whitney U (or Kruskal–Wallis) test and Pearson’s chi-square test (or Fisher’s exact test as appropriate) for continuous and categorical variables, respectively. Comparisons between time variables (i.e., days spent in ICU and days between admission and VD administration) were assessed using Poisson regression models. A two-sided *p* value <0.05 was considered statistically significant. All statistical analyses were performed using SAS Release 9.4 (SAS Institute, Cary, NC, USA).

## Results

A total of 132 were screened and 73 patients (42 M and 31 F; mean age 53.2 ± 15.7) were included in the trial (CONSORT flow diagram). Of these, 36 (21 M and 15F; mean age 57.52 ± 14.88) were randomized to the VD supplementation group (VDsG) and 37 (20 M and 17 F; mean age 48.28 ± 17.47) to the CG. Intent-to-treat analysis was performed. All patients were of Caucasian origin, representative of the national population. No statistical difference was detected between the two groups for demographic and clinical features, apart from the age that was significantly higher in the VDsG than the CG (*p* = 0.026). The causes of cerebral damage were hemorrhage (30%), brain injury (26%), ischemic stroke (12.3%), cerebral anoxia (2.7%), meningioma (4.1%), and encephalitis (2.7%). At admission in neurorehabilitation, all patients showed severe clinical and neurological pictures (ill conditions). All subjects breathed spontaneously but required various levels of oxygen therapy and had tracheal tube. Furthermore, all subjects showed severe dysphagia treated by nasogastric tube (NGT) or PEG. Demographic characteristics of the whole sample and of the VDsG and CG are reported in Table 1. During the neurorehabilitation stay, almost all subjects suffered from sepsis and infections involving urinary, respiratory tract, and skin wounds that required multiple antibiotic drugs. The presence of multiple bacterial species has been so far the most frequent, including multiple drug-resistant germs, in particular *Acinetobacter baumannii*, *Enterobacteriaceae*, *Pseudomonas aeruginosa*, and *Staphylococcus aureus*.

**Table 1.** Demographics and Clinical Characteristics of Enrolled sABI Patients

	CG (*n* = 37)	VDsG (*n* = 36)	Total (*n* = 73)	*p*-Value
Sex				1.000
F	16 (43.2%)	15 (41.7%)	31 (42.5%)	
M	21 (56.8%)	21 (58.3%)	42 (57.5%)	
Age				0.029
Mean (SD)	49.60 (15.46)	57.52 (14.88)	53.51 (15.59)	
Median (Q1, Q3)	49.06 (41.40, 60.73)	58.92 (48.69, 67.27)	55.52 (45.51, 65.85)	
Min–Max	17.48–80.03	20.87–82.28	17.48–82.28	
Etiology				0.821
Cerebral hemorrhage	14 (37.8%)	16 (44.4%)	30 (41.1%)	
Traumatic brain injury	16 (43.2%)	11 (30.6%)	27 (37.0%)	
Cerebral ischemia	4 (10.8%)	6 (16.7%)	10 (13.7%)	
Cerebral anoxia	1 (2.7%)	1 (2.8%)	2 (2.7%)	
Neoplasm	1 (2.7%)	2 (5.6%)	3 (4.1%)	
Encephalitis	1 (2.7%)	0 (0.0%)	1 (1.4%)	
LOS ICU				0.656
Mean (SD)	33.27 (24.68)	35.44 (15.74)	34.34 (20.64)	
Median (Q1, Q3)	26.00 (22.00, 36.00)	31.00 (25.25, 42.00)	29.00 (22.00, 39.00)	
Min – Max	11.00–150.00	13.00–76.00	11.00–150.00	
ACUR	6 (16.2%)	4 (11.1%)	10 (13.7%)	0.607
Dead	1 (2.7%)	3 (8.3%)	4 (5.5%)	
LOS NR				0.404
Mean (SD)	59.49 (37.35)	66.75 (36.54)	63.07 (36.88)	
Median (Q1, Q3)	50.00 (34.00, 74.00)	63.00 (35.25, 98.50)	57.00 (34.00, 92.00)	
Min–Max	9.00–150.00	3.00–135.00	3.00–150.00	

ACUR, acute care unit readmission; CG, control group; ICU, intensive care unit; LOS, length of stay; NR, neurorehabilitation setting; sABI, severe acquired brain injury; SD, standard deviation; VDsG, vitamin D supplementation group.

## Functional Outcome

**FIG. 1.**
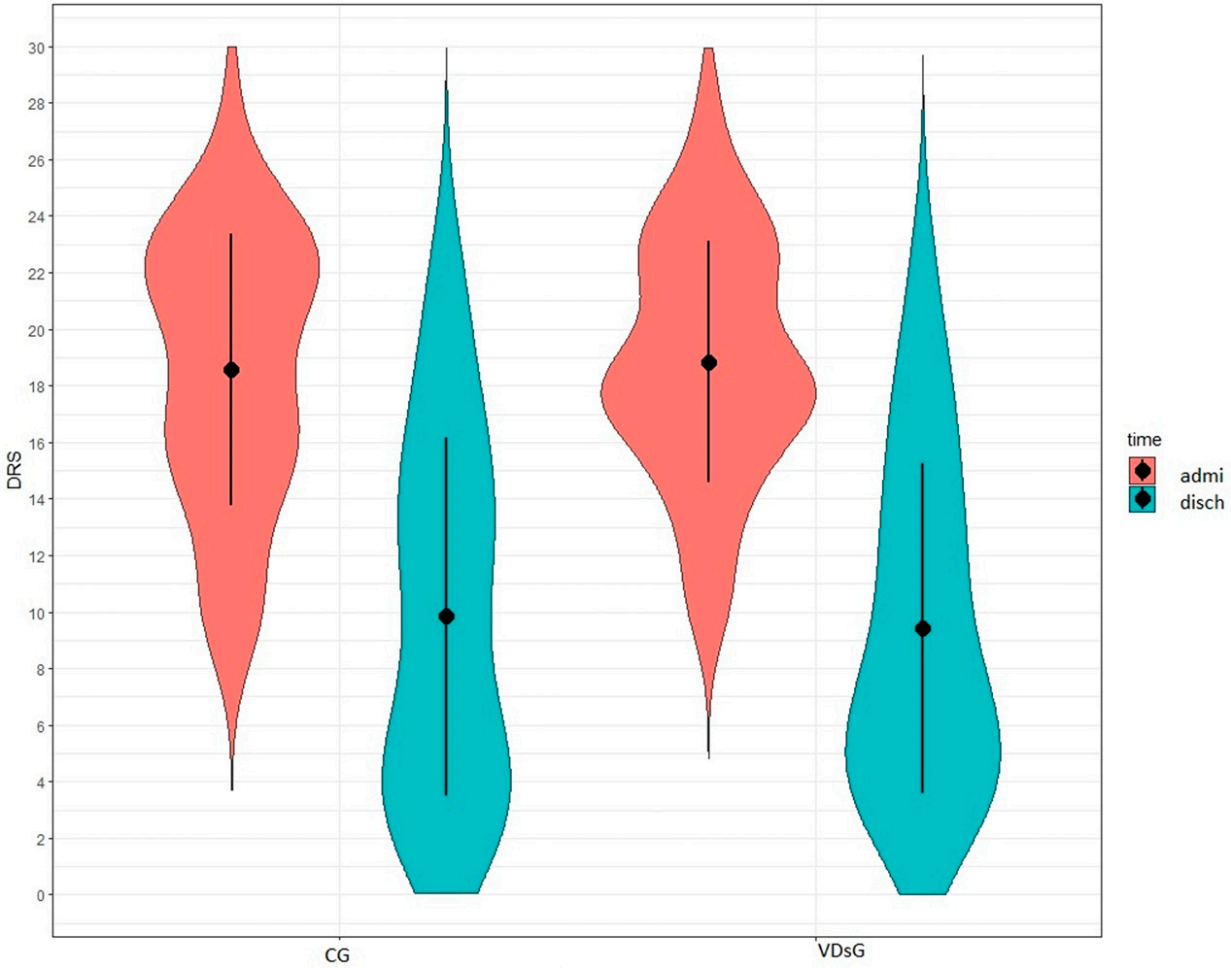
Disability Rating Scale in enrolled sABI patients at admission and at discharge. DRS, Disability Rating Scale; CG, control group; VDsG, vitamin D supplementation group.

**FIG. 2.**
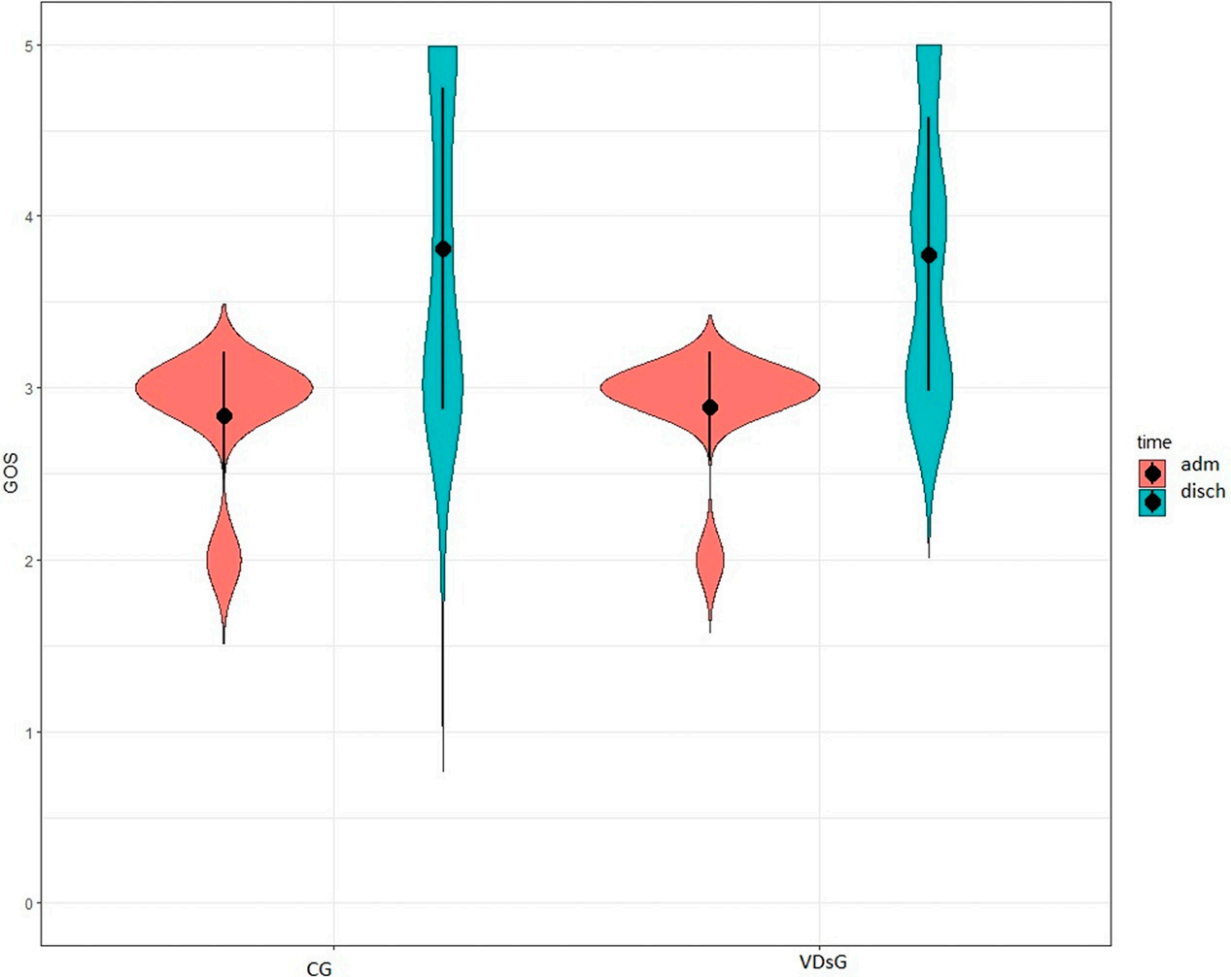
Glasgow Outcome scale in enrolled sABI patients at admission and at discharge. GOS, Glasgow Outcome Scale; CG, control group; VDsG, vitamin D supplementation group.

**FIG. 3.**
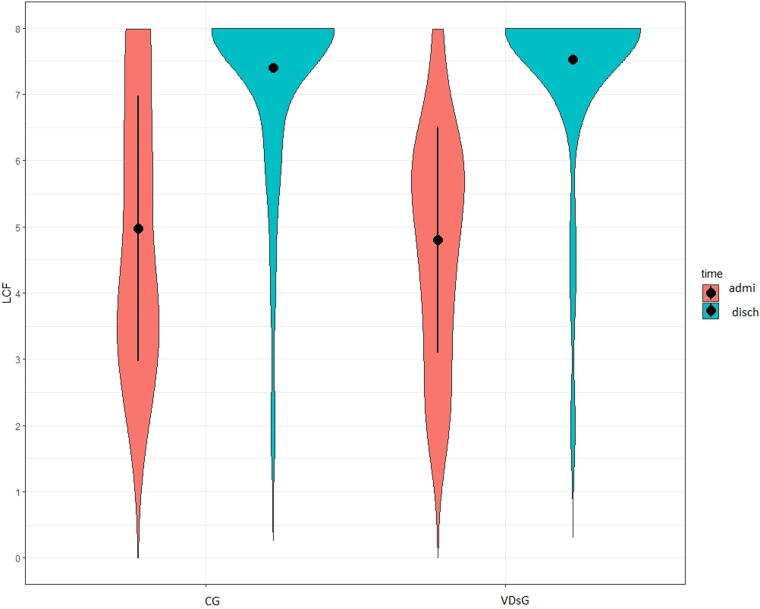
Level of Cognitive Functioning in enrolled sABI patients at admission and at discharge. LCF, Level of Cognitive Functioning; CG, control group; VDsG, vitamin D supplementation group.

At admission, the functional status did not show any differences between the two groups, as shown in the DRS, GOS, and LCF scores (Table 2). At discharge, all patients showed significant functional improvement. DRS significantly decreased both in VDsG: mean −9.42 ± 4.30 (*p* < 0.001) and in CG: mean −8.73 ± 5.28 (*p* < 0.001) but without statistical significance (*p* = 0.564) (Table 2, Fig. 1). After adjusting for patients’ age, this difference remained not statistically different (*p* = 0.549). GOS increased in VDsG: mean 0.89 ± 0.85 (*p* < 0.001) and in CG: mean 0.97 ± 0.90 (*p* < 0.001), and the changes did not result in statistical difference (*p* = 0.683) (Table 2, Fig. 2) as well as after adjusting for patients’ age (*p* = 0.785). Likewise, LCF increased both in VDsG: mean 2.72 ± 1.80 (*p* < 0.001) and in CG: mean 2.43 ± 2.15 (*p* < 0.001). The changes did not result in statistical difference (*p* = 0.535, Table 2, Fig. 3). After adjusting for patients’ age, this difference remained not statistically different (*p* = 0.812). No difference was detected in the functional outcome when stratifying patients for sufficient, insufficient, and deficient VD serum level. However, in the VDsG subjects with deficiency of VD, the differences of score values of DRS and GOS were higher than those of CG: −10.05 ± 4.81 and −7.86 ± 4.80 for DRS; 0.95 ± 0.80 and 0.86 ± 0.77 for GOS in VDsG and CG, respectively (Supplementary Appendix S1).

**Table 2.** Functional Scale Score of Enrolled Subjects with sABI

	CG (*n* = 37)	VDsG (*n* = 36)	Total (*n* = 73)	*p*-Value
DRS- admission				0.804
Mean (SD)	18.57 (4.80)	18.83 (4.27)	18.70 (4.52)	
Median (Q1, Q3)	18.00 (15.00, 22.00)	18.00 (16.75, 22.00)	18.00 (16.00, 22.00)	
Min–Max	10.00–28.00	10.00–28.00	10.00–28.00	
DRS discharge				0.769
Mean (SD)	9.84 (6.34)	9.42 (5.83)	9.63 (6.05)	
Median (Q1, Q3)	8.00 (3.00, 15.00)	8.00 (4.75, 14.00)	8.00 (4.00, 14.00)	
Min–Max	2.00–22.00	3.00–22.00	2.00–22.00	
DRS difference				0.564
Mean (SD)	−8.73 (5.28)	−9.42 (4.83)	−9.07 (5.04)	
Median (Q1, Q3)	−8.00 (−13.00, −6.00)	−10.00 (−13.00, −6.75)	−10.00 (−13.00, −6.00)	
Min–Max	−16.00 to 6.00	−17.00 to 0.00	−17.00 to 6.00	
GOS admission				0.533
Mean (SD)	2.84 (0.37)	2.89 (0.32)	2.86 (0.35)	
Median (Q1, Q3)	3.00 (3.00, 3.00)	3.00 (3.00, 3.00)	3.00 (3.00, 3.00)	
Min-Max	2.00–3.00	2.00–3.00	2.00–3.00	
GOS-discharge				0.872
Mean (SD)	3.81 (0.94)	3.78 (0.80)	3.79 (0.87)	
Median (Q1, Q3)	4.00 (3.00, 5.00)	4.00 (3.00, 4.00)	4.00 (3.00, 5.00)	
Min–Max	2.00–5.00	3.00–5.00	2.00–5.00	
GOS difference				0.683
Mean (SD)	0.97 (0.90)	0.89 (0.85)	0.93 (0.87)	
Median (Q1, Q3)	1.00 (0.00, 2.00)	1.00 (0.00, 1.25)	1.00 (0.00, 2.00)	
Min–Max	−1.00 to 2.00	0.00–3.00	−1.00 to 3.00	
LCF admission				0.702
Mean (SD)	4.97 (2.01)	4.81 (1.70)	4.89 (1.85)	
Median (Q1, Q3)	4.00 (3.00, 6.00)	5.00 (3.75, 6.00)	5.00 (3.00, 6.00)	
Min–Max	2.00–8.00	2.00–8.00	2.00–8.00	
LCF discharge				0.689
Mean (SD)	7.41 (1.32)	7.53 (1.28)	7.47 (1.29)	
Median (Q1, Q3)	8.00 (8.00, 8.00)	8.00 (8.00, 8.00)	8.00 (8.00, 8.00)	
Min–Max	2.00–8.00	2.00–8.00	2.00–8.00	
LCF difference				0.535
Mean (SD)	2.43 (2.15)	2.72 (1.80)	2.58 (1.98)	
Median (Q1, Q3)	3.00 (1.00, 4.00)	2.00 (2.00, 4.00)	2.00 (1.00, 4.00)	
Min–Max	−4.00 to 6.00	0.00–6.00	−4.00 to 6.00	

CG, control group; DRS, Disability Rating Scale; GOS, Glasgow Outcome Scale; LCF, Level of Cognitive Functioning; sABI, severe acquired brain injury; SD, standard deviation; VDsG, vitamin D supplementation group.

### VD serum level

At baseline, almost all patients had low serum level of VD, and it was not different between the two groups: 21.27 ± 10.32 ng/mL and 22.18 ± 7.51 ng/mL in VDsG and CG, respectively. No difference was detected between the two groups for number of subjects stratified in sufficient, insufficient, and deficient serum levels of VD. At discharge, serum levels of VD decreased in CG (mean −1.91 ± 4.26, *p* = 0.066). On the contrary, a significant increase was detected in VDsG (mean 3.88 ± 6.90, *p* = 0.02), and these changes resulted in statistical difference (*p* = 0.003, Table 3). After adjusting for patients’ age, this difference remained statistically different (*p* = 0.004). Although the VD serum level increased in VDsG, the mean value remained at an insufficient level: 23.73 ± 6.54 ng/mL.

**Table 3.** Vitamin D Serum Level and Stratification in sABI

	CG (*n* = 37)	VDsG (*n* = 36)	Total (*n* = 73)	*p*-Value
VD admission	ng/mL	ng/mL	ng/mL	
Mean (SD)	22.18 (7.51)	21.27 (10.32)	21.73 (8.96)	0.668
Median (Q1, Q3)	21.20 (17.60, 27.30)	18.45 (13.88, 25.62)	20.10 (14.50, 27.30)	
Min–Max	6.50–46.00	8.20–49.90	6.50–49.90	
VD discharge	ng/mL	ng/mL	ng/mL	
Mean (SD)	18.59 (6.84)	23.73 (6.54)	21.23 (7.09)	0.022
Median (Q1, Q3)	18.90 (14.95, 22.70)	23.20 (18.27, 27.92)	20.40 (17.80, 26.00)	
Min–Max	6.10–31.30	10.80–38.90	6.10–38.90	
VD difference				
Mean (SD)	−1.91 (4.26)	3.88 (6.90)	1.06 (6.40)	0.003
Median (Q1, Q3)	−2.60 (−4.70, 1.10)	2.60 (−0.83, 9.63)	0.80 (−3.35, 3.95)	
Min–Max	−10.50 to 7.60	−6.70 to 16.90	−10.50 to 16.90	
VD strat. admis.	N	N	N	0.176
Sufficient	5 (13.5%)	5 (13.9%)	10 (13.7%)	
Insufficient	18 (48.6%)	10 (27.8%)	28 (38.4%)	
Deficient	14 (37.8%)	21 (58.3%)	35 (47.9%)	
VD strat. disch.				0.141
Sufficient	1 (5.3%)	3 (15.0%)	4 (10.3%)	
Insufficient	6 (31.6%)	11 (55.0%)	17 (43.6%)	
Deficient	12 (63.2%)	6 (30.0%)	18 (46.2%)	

CG, control group; N, number; sABI, severe acquired brain injury; SD, standard deviation; VD, vitamin D; VDsG, vitamin D supplementation group.

## Discussion

Oral supplement of VD did not improve the functional outcome of sABI in subjects who underwent intensive neurorehabilitation. All patients significantly improved and no difference was observed between VDsG and the CG, after rehabilitation. However, although not statistically significant, patients in VDsG who had VD deficiency at admission showed a tendency to increase the values of the scores on DSR and GOS than those of the same subgroup in CG.

Studies have demonstrated that VD has a neuroprotective and an anti-inflammatory effect and that supplementation can produce benefits in subjects suffering from several neurological diseases, particularly in MS and ischemic stroke. Concerning that point, a low VD status could predict a higher risk of exacerbations and magnetic resonance imaging activity in people with early RRMS. In these patients, each 25 nmol/L (10 ng/L) increase in VD could produce 14–15% reduction of the risk of subsequent relapses and 15–50% reduction of the risk of new or enlarging T2 and/or gadolinium-enhancing T1 lesions on magnetic resonance imaging.^27^ However, although observational studies have showed that VD supplementation has significant positive effects in RRMS, large randomized studies have been negative when primary clinical end-points were no evidence of disease activity^27^ and annualized relapse rate.^28^ Likewise, systematic reviews on this issue were not conclusive^29,30^ and suggested that the supplementation may have a therapeutic action in the treatment of MS even if there is uncertainty with regard to the most appropriate dose. Hence, the effects of VD supplementation in MS remain unclear. In the same way, a number of studies have observed that VD deficiency can be a significant risk factor for ischemic stroke and might have a negative prognostic value. On the contrary, although recent systematic reviews confirmed that lower circulating levels of VD were associated with an elevated risk of stroke, they did not confirm that extra VD supplementation reduced major adverse cardiovascular events and the risk of stroke.^31,32^ Stroke is a leading cause of disability; hence, it is worth striving for new therapeutic approaches in addition to rehabilitative strategies to ameliorate the outcome of poststroke subjects. Randomized studies investigating the role of VD supplementation in the recovery of these subjects have demonstrated contrasting findings. Gupta et al. detected that VD supplementation had a beneficial effect in improving the outcome.^16^ Likewise, Sari et al. found that subjects suffering from stroke treated with VD supplementation during rehabilitation increased the activity levels and accelerated balance recovery, even if the patients did not show a significant improvement of ambulation or motor recovery.^17^ Conversely, Torrisi et al.^33^ detected that the beneficial effect on functional recovery was mainly due to rehabilitation rather than VD supplementation.

In the present study, we have not found any benefits of VD supplementation in improving the outcome of subjects with sABI, and the findings are in line with those of studies investigating the effects of VD supplementation in critically ill patients during their ICU stay. In this respect, patients with sABI show severe clinical pictures resembling those of critically ill patients. All of our samples came from the ICU, and all had care devices such as tracheal tube, NGT, or PEG. Furthermore, about all patients had complications, in particular various infections and sepsis that required multiple antibiotic drugs. Karsy et al.^34^ have recently performed a randomized study to investigate the effect of VD supplementation in neurocritical patients. They evaluated whether VD supplementation would result in a clinically meaningful reduction in hospital length of stay (LOS). The study included only subjects with VD deficiency levels ≤20 ng/mL. The patients who were randomized to VD supplementation received VD3 (cholecalciferol) at the dose of 540.000 IU orally or placebo. A total of 134 patients underwent VD3 treatment compared with 133 control patients. The study did not find differences between the groups and did not detect the benefits of VD supplementation in hospital or ICU LOS as well as in complications (e.g., sepsis, cardiovascular, ventilation use, hyperglycemia, and other). A similar finding has been reported in a previous study enrolling critical not neurological ICU subjects.^35^ Therefore, in critically ill patients, although previous studies have demonstrated that VD deficiency is predictive of worse outcomes,^36,37^ VD supplementation did not improve the clinical course but produced only the decrease of inflammatory biomarkers such as interleukin (IL)-6, Il-10, and C-protein reactive.^38,39^ However, biomarkers of the disease may be expression of inflammation and do not necessarily reflect a clinically meaningful course.

So far, the effects of VD supplementation remain unclear, and this aspect could be due to several reasons. In this regard, clinical trials have remained limited and few randomized studies enrolling small samples have been conducted. Furthermore, a different method design has been used, including characteristics of population, aim, VD dosage, modality of administration, duration of treatment, and follow-up. Important questions concern the amount of VD supplementation that should be administered, route of administration (oral, intramuscular, or intravenous), time (single or daily dose), and duration of treatment. We used an initial oral dose of 50.000 UI and then a daily VD amount of 1.000 UI. This dosage was lower than the amount used in studies that investigated critically ill patients, and this dosage might have been not sufficient to reach normal serum levels to obtain potential benefits on the recovery. At discharge, the mean serum value of VDsG remained at an insufficient level: 23.73 ± 6.54 ng/mL. However, studies that investigated a similar population, although they used a higher dose of VD, did not find beneficial effects with supplementation in the critically ill population.^34,35^ Likewise, in poststroke subjects who underwent VD supplementation, a widely variable amount and route of VD administration were used, including single intramuscular doses ranging from 100.000 to 600.000 UI,^16,22^ oral single dose of 50.000 UI, or daily dose of 2000 UI. Therefore, the variability of doses and modalities of treatment do not permit to compare the studies; hence, the proper amount of VD supplementation in obtaining a potential beneficial effect remains unclear. Moreover, further issues remain unsolved and limit the finding on VD supplementation and require future investigations—for example, the role of genetic factors and the association of calcium. Indeed, recent evidence suggests an underlying genetic role in the relationship between VD deficiency and some neurological diseases, in particular MS^3,40^ and stroke.^41,42^ Calcitriol, the active form of VD, affects gene expression, both directly and indirectly through the nuclear VD receptor.^43,44^ Likewise, the role of calcium association to VD supplementation has been suggested but remains unsolved.^45^

A further reason might be the lack or the limitations of the measures used to investigate the outcome in sABI subjects. Different measurements and indicators, including mortality, genetic factors, and scales focusing on specific functional domains, might be useful and should be considered in future studies.

## Limitations

The present study has limitations, including the small number of samples and the lack of follow-up. Some patients in both groups during the rehabilitation were readmitted to the acute care unit following complications such as respiratory failure and sepsis, but we performed an intention-to-treat analysis that included also the patients who withdrew from the trials. As all subjects were fed by PEG or NGT, it is not possible to exclude that VD supplementation was not completely administered because of removal or dysfunction of devices and to potential interference with nourishment or drugs. Furthermore, the study was conducted in a single center, limiting the generalization of the finding.

## Conclusion

VD supplementation did not produce any improvements in functional outcomes in sABI during the intensive rehabilitation treatment. However, sABI subjects of VDsG with deficiency of VD showed mean higher scores to DRS and GOS compared with those without VD supplementation, after rehabilitation. The effect of VD supplementation on recovery of the sABI remains unclear and future studies with a large sample, a higher VD dosage, and a longer follow-up are needed.

## Supplementary Material

Supplementary Appendix S1

## Abbreviations Used

ACUR: Acute care unit readmission
CG: Control group
DRS: Disability Rating Scale
GOS: Glasgow Outcome Scale
ICU: Intensive care unit
LCF: Level of Cognitive Functioning
LOS: Length of stay
NGT: Nasogastric tube
NR: Neurorehabilitation setting
PEG: Percutaneous endoscopic gastrostomy
RRMS: Relapsingremitting MS
sABI: Severe acquired brain injury
SD: Standard deviation
VD: Low vitamin D
VDsG: Vitamin D supplementation group
